# Comparative Transcriptomics Reveals Discrete Survival Responses of *S. aureus* and *S. epidermidis* to Sapienic Acid

**DOI:** 10.3389/fmicb.2017.00033

**Published:** 2017-01-25

**Authors:** Josephine C. Moran, Jamal A. Alorabi, Malcolm J. Horsburgh

**Affiliations:** Functional and Comparative Genomics, Institute of Integrative Biology, University of LiverpoolLiverpool, UK

**Keywords:** RNA-Seq, *Staphylococcus aureus*, *Staphylococcus epidermidis*, sapienic acid, fatty acid, skin, colonization

## Abstract

Staphylococcal colonization of human skin is ubiquitous, with particular species more frequent at different body sites. Whereas *Staphylococcus epidermidis* can be isolated from the skin of every individual tested, *Staphylococcus aureus* is isolated from <5% of healthy individuals. The factors that drive staphylococcal speciation and niche selection on skin are incompletely defined. Here we show that *S. aureus* is inhibited to a greater extent than *S. epidermidis* by the sebaceous lipid sapienic acid, supporting a role for this skin antimicrobial in selection of skin staphylococci. We used RNA-Seq and comparative transcriptomics to identify the sapienic acid survival responses of *S. aureus* and *S. epidermidis*. Consistent with the membrane depolarization mode of action of sapienic acid, both species shared a common transcriptional response to counteract disruption of metabolism and transport. The species differed in their regulation of SaeRS and VraRS regulons. While *S. aureus* upregulated urease operon transcription, *S. epidermidis* upregulated arginine deiminase, the oxygen-responsive NreABC nitrogen regulation system and the nitrate and nitrite reduction pathways. The role of *S. aureus* ACME and chromosomal arginine deiminase pathways in sapienic acid resistance was determined through mutational studies. We speculate that ammonia production could contribute to sapienic acid resistance in staphylococci.

## Introduction

Staphylococci are major commensal colonizers of healthy human skin and leading causes of hospital-acquired infections, responsible predominantly for wound and device-associated infections. Understanding skin survival mechanisms of staphylococci is vital, as individuals colonized by *Staphylococcus aureus* are at greater risk of infections during hospitalization (Kluytmans et al., 1997; Davis et al., 2004).

Unlike those coagulase-negative staphylococci that are skin-dwelling, the primary human niche of coagulase-positive *S. aureus* is the nares, with skin colonization being transient and seeded from this location (Moss and Squire, 1948; Kluytmans and Wertheim, 2005; Cho et al., 2010). A comparison of responses and resistance mechanisms between *S. aureus* and these closely related, long-term skin colonizers, such as *S. epidermidis*, therefore provides a useful tool to investigate functionalities required for skin colonization and persistence (Coates et al., 2014). Such investigations have increasing relevance with the emergence of community-acquired MRSA lineages, such as USA300 which cause increased skin pathology (Moran et al., 2006; Li et al., 2009).

Atopic dermatitis is a disease presenting as dry, flaky skin lesions, abscesses, and unusually high levels of *S. aureus* skin colonization (Higaki et al., 1999; Bieber, 2008; Kong et al., 2012). Many host factors of skin are altered in atopic dermatitis, including levels of antimicrobial peptides, antimicrobial fatty acids, and sphingosines, all of which have been associated with *S. aureus* exclusion (Schafer and Kragballe, 1991; Arikawa et al., 2002; Cho et al., 2010). Levels of sapienic acid in particular were determined to be inversely proportional to levels of *S. aureus* (Takigawa et al., 2005), identifying sapienic acid as a strong candidate host factor that contributes to prevention of long-term skin colonization by *S. aureus*.

Recent studies revealed the effects that sapienic acid and other skin fatty acids have on *S. aureus* survival (Kenny et al., 2009; Cartron et al., 2014; Neumann et al., 2015). Together these studies showed that unsaturated long-chain fatty acids, including sapienic and linoleic acids, cause membrane depolarization in *S. aureus* leading to large transcriptional changes, especially those pathways associated with cellular energetics (Kenny et al., 2009; Neumann et al., 2015). From the transcriptomic response, it is inferred that the membrane depolarisation leads to disruption of the electron transport chain (Kenny et al., 2009; Neumann et al., 2015).

Here we show that the mean sapienic acid MIC of *S. epidermidis* strains is greater than *S. aureus*. Consequently, RNA-Seq was used to compare sapienic acid transcriptional responses with the aim of highlighting skin survival determinants. These investigations form the basis to determine whether sapienic acid responses discriminate staphylococcal species based on their skin-dwelling propensity.

## Materials and Methods

### Bacterial Strains and Culture

Strains used in this study are listed in **Table 1**. Overnight cultures were grown for 18 h at 37°C with shaking. Todd Hewitt broth (THB) or agar (THA) was used as the culture media for all experiments. Sapienic acid (Matreya) stock solution was prepared at 8 mg ml^-1^ in ethanol. Antibiotics were incorporated at concentrations of 12.5 μg ml^-1^ tetracycline, 100 μg ml^-1^ ampicillin, 10 μg ml^-1^ chloramphenicol, and 5 μg ml^-1^ erythromycin, when appropriate.

**Table 1:** Strains used in this study.

Species	Strain	Description	Reference
*S. epidermidis*	Rp62a	Intravascular catheter isolate	Christensen et al., 1982, 1985
	Tü3298	Epidermin producer	Allgaier et al., 1986
	NCTC 1457	PIA producer	Mack et al., 1992
	A19	Recent skin (forearm) isolate	Kelly, 2013
	B19	Recent skin (forearm) isolate	Kelly, 2013
	O16	Recent skin (forearm) isolate	Kelly, 2013
	BL115	Recent nasal isolate	Libberton, 2011
*S. aureus*	Newman	Osteomyelitis isolate	Duthie and Lorenz, 1952
	SH1000	Lab strain (rsbU repaired 8325-4 derivative)	Horsburgh et al., 2002
	MSSA476	Osteomyelitis isolate	Holden et al., 2004
	MRSA252	Fatal bacteraemia isolate, MRSA	Holden et al., 2004
	BL014	Recent nasal isolate	Libberton, 2011
	BL032	Recent nasal isolate	Libberton, 2011
	SF8300	CA-MRSA	Diep et al., 2008
	SF8300ax	SF8300 with ACME deletion	Diep et al., 2008
	Liv1245	Newman arcA::tet from Liv692	This study
	Liv1247	SF8300 arcA::tet from Liv692	This study
	Liv1249	SF8300ax arcA::tet from Liv692	This study
	Liv692	S. aureus SH1000 arcA::tet	Kenny et al., 2009
	Newman tagO	tagO::ery from SA113 tagO	This study
	SA113 tagO	tagO::Ery	Bera et al., 2005
	Newman *mcrA*	NWMN_0050:: Ery	This study
	Newman *mcrA* pSK5632	Newman *mcrA* containing pSK5632 + *mcrA*	This study
	Liv1023	*mtlD*::*tet* (SH1000)	Kenny et al., 2013
	Liv1024	*mtlABCD*::tet (SH1000)	Kenny et al., 2013
	RN4220	Restriction deficient strain	Kreiswirth et al., 1983
	SH1000 *mnhF*	*mnhF* in frame unmarked deletion	Sannasiddappa et al., 2015


### Minimum Inhibitory Concentration Assay

Minimum inhibitory concentration (MIC) assays were performed using a broth microdilution method in 96 well plates, with final well volumes of 200 μl and a sapienic acid concentration range of 200–0.8 μg ml^-1^. An inoculum of ∼10^4^ CFU ml^-1^ was used.

### Growth and Sapienic Acid Challenge

Overnight broth cultures were adjusted to an OD_600_ of 0.5 then diluted 25-fold in fresh medium prior to incubation in a water bath with shaking (250 rpm) at 37°C. Sapienic acid/ethanol was added to cell cultures in mid-exponential phase (OD_600_ ∼0.5) with equivalent volumes of ethanol added to control cultures. For RNA-Seq experiments, cells were harvested by centrifugation 20 min after challenge and suspended in RNA*later* (Qiagen).

### RNA Extraction and Library Preparation

For cell lysis, bacteria were pelleted at 6,000 RCF for 5 min at 4°C and suspended in 100 μl TE containing 6 mg ml^-1^ lysostaphin and 400 U ml^-1^ mutanolysin. Lysis was performed for 15 min at 37°C for *S. aureus* and 30 min for *S. epidermidis*. Subsequently, samples were treated with 25 μl of proteinase K (Qiagen) for 30 min at 37°C. RNA was extracted using the RNeasy kit (Qiagen). Samples were DNase-treated using turbo DNase (Ambion), and the DNase removed using the RNeasy MinElute clean up kit (Qiagen).

Depletion of rRNA was achieved with a Ribo-Zero magnetic kit for Gram-positive bacteria (Epicentre). The concentration of RNA was normalized before library construction using strand specific ScriptSeq kits (Epicentre); libraries were prepared by the Centre for Genomic Research (CGR), Liverpool. RNA-Seq samples were sequenced by paired-end sequencing using the HiSeq platform (Illumina).

### RNA-Seq Differential Expression Analysis

Bowtie (Langmead et al., 2009) and Edge R (Robinson and Oshlack, 2010; Robinson et al., 2010) were used to map reads and determine the differentially expressed (DE) genes, respectively. Genes with mapped transcripts that had a false discovery rate <0.05, as determined by Benjamin and Hochberg analysis, were considered differentially expressed between control and test conditions.

Gene expression changes in biosynthetic pathways were associated using KEGG mapper-search and color (Kanehisa and Goto, 2000; Kanehisa et al., 2012). The *S. aureus* transcriptome meta-database (SATMD) (Nagarajan and Elasri, 2007) was used to compare sapienic acid DE gene sets with existing *S. aureus* DE gene sets.

### cDNA Generation and qPCR

The tetro cDNA synthesis kit (Bioline) was used for cDNA synthesis using random hexamer primers and 2 μg RNA per reaction. **Table 2** lists the qPCR primers. Novel primers were designed using primer-BLAST (Ye et al., 2012). Primer efficiency for all primers was confirmed to be within 90–100% as described previously (Nolan et al., 2006). All qPCR reactions were performed using SensiFAST SYBR Hi-ROX kit (Bioline) with the ABI StepOnePlus (Life Technologies); data analysis used the ABI StepOnePlus software. At least two technical replicates and three biological replicates were used to determine fold change in gene expression between samples.

**Table 2:** Primers used in this study.

Gene name	Primer sequences	Efficiency (%)	Reference
*rpoB*	F-GCGAACATGCAACGTCAAGR-GACCTCTGTGCTTAGCTGTAATAGC	97.0	This study
*hu*	F-TTTACGTGCAGCACGTTCACR-AAAAAGAAGCTGGTTCAGCAGTAG	90.3	Duquenne et al., 2010
*gyrB* Tü3298	F-AGAAAAGATGGGACGCCCTGR-CACCATGAAGACCGCCAGAT	96.6	This study
*gyrB* Newman	F-ATCGACTTCAGAGAGAGGTTGR-CCGTTATCCGTTACTTTAATCCA	92.9	Kenny et al., 2009
*capB*	F-GCGATATGCGTAAGCCAACACR-GGTACAGGGCCAGCTGTTAG	91.5	This study
*pyrP*	F-CGATGTTTGGCGCAACAGTAR-GCTGGTATTTGCGCCTTCG	92.5	This study
*clpB*	F-TGGTGCACCTCCAGGTTATGR-AGAATCCGTAAGACGACCTTCA	99.0	This study
*farR*	F-ACGCCAGCTGTGTGGATTATR-AACGACTGCGACCTTGATGT	93.3	This study
*sasF*	F-TCACTCTGCGATTGAAGGCAR-TTTCCGGTGCCGAATGATCT	95.0	This study
*narH*	F-TGGCCTTTCCATTGCATCCTR-TTCAGTGTCGCCAGCAGTTA	93.6	This study
*mcrA* (gene knockout)	F1-ACATGAATTCGGAATTGGTTAAGTTCACTCR1-CCGGTACCAGAACTCATCTAATA CAGACF2-ATAACTGCGGCCGCTGTATCACTTAGGTGTATCAR2-CGACGGATCCTCCAGCTGTTACCAGTCCGA	–	This study
*mcrA* (complementation)	F-TTACGGATCCTTAAGTAACTTCTTTCAAR-TTATAAAGCTTACATCATTTCTGTCCCAG	–	This study


### Construction of Gene Mutants

An allelic replacement mutant of NWMN_0050 and complementation of this mutant were constructed using the previously described method of Horsburgh et al. (2004) using the primers listed in **Table 2**. Allelic replacement mutants of *tagO* and *arcA* in strain Newman were generated by phage transduction (Horsburgh et al., 2001) from previously described mutants (**Table 1**).

### Data Accession Numbers

The complete genome sequence of *S. epidermidis* Tü3298 is available at http://www.ebi.ac.uk/ena/data/view/PRJEB11651 (Moran and Horsburgh, 2016). The Illumina sequence read data generated from the RNA-Seq experiments are available from ArrayExpress database^1^ under accession number E-MTAB-4587.

## Results and Discussion

### Comparative Sapienic Acid Resistance

**FIGURE 1:**
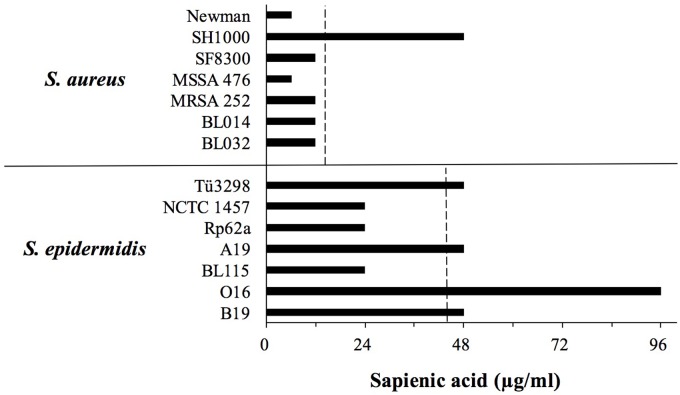
**Sapienic acid minimum inhibitory concentration (MICs) of *S. aureus* and *Staphylococcus epidermidis* strains.** MICs were determined using the broth microdilution method. MICs are the lowest concentration found to inhibit growth. The dotted line shows the mean MIC of each species.

We hypothesized that differences in sapienic acid resistance might contribute to the higher frequency and persistence of *S. epidermidis* on healthy human skin relative to *S. aureus*. Consistent with our hypothesis, we identified that the mean sapienic acid MIC of *S. epidermidis* was approximately three times higher than that of *S. aureus* strains (*p* = 0.023) (**Figure 1**).

Previous studies revealed that *S. aureus* colonizes the skin of atopic dermatitis sufferers, and its colonization frequency inversely correlates with sapienic acid levels (Takigawa et al., 2005). Sapienic acid is not the only factor on atopic skin to be linked with *S. aureus* colonization, and levels of other skin lipids and antimicrobial peptides are also linked with colonization (Schafer and Kragballe, 1991; Arikawa et al., 2002; Cho et al., 2010). Although sapienic acid is antimicrobial it remains to be demonstrated that within sebum its activity *in vivo* reduces *S. aureus* colonization. These *in vitro* data presented here support a hypothesis that sapienic acid resistance contributes to skin colonization and persistence. The differential survival data are consistent with the increased frequency of staphylococci, not only *S. aureus*, on skin of atopic dermatitis patients whose skin lipid levels are reduced (Kong et al., 2012; Soares et al., 2013).

While *S. epidermidis* strains exhibited a greater sapienic acid MIC compared with *S. aureus*, *S. epidermidis* growth was inhibited by lower concentrations than skin colonizing corynebacteria. For example, *Corynebacterium stratum* which colonizes sebaceous niches has up to 10 times greater sapienic acid MIC (Fischer et al., 2012) than the *S. epidermidis* strains studied here.

### Growth of Sapienic Acid-Challenged *S. aureus* and *S. epidermidis*

Similar to findings of our previous study of the *S. aureus* response to linoleic acid (Kenny et al., 2009), sapienic acid was reported by Neumann et al. (2015) to induce a major adaptive transcriptional response in *S. aureus* SH1000. We therefore hypothesized that comparison of the transcriptional responses of *S. aureus* with *S. epidermidis* might identify differential skin survival mechanisms of these species relating to antimicrobial lipids that might also account for their MIC differences. Since we were interested in the typical response of each species, strains *S. aureus* Newman and *S. epidermidis* Tü3298 were selected, being representative of each species based on their MICs.

**FIGURE 2:**
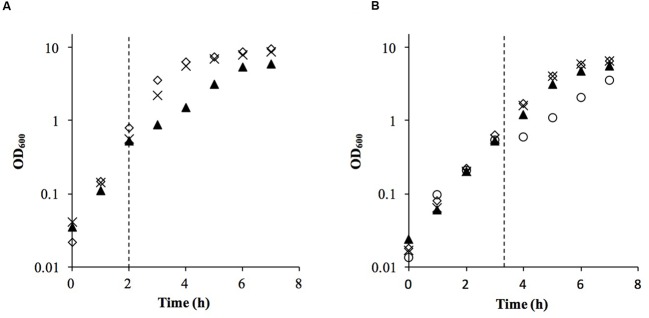
**Growth of *S. aureus* Newman** **(A)** and *S. epidermidis* Tü3298 **(B)** challenged with sapienic acid. Bacteria were grown to OD_600_ = 0.5 before addition of sapienic acid (dotted line). Sapienic acid was added to concentrations 7.5 μM (cross), 11.25 μM (triangle) and 15 μM (circle)-Tü3298 only- or no addition, 0 μM (diamond).

We used a similar experimental design to our previous linoleic acid transcriptional response study (Kenny et al., 2009), substituting microarrays with RNA-Seq for transcriptomics. Both staphylococci were grown to mid-log phase and challenged with the lowest concentration of sapienic acid that caused an equivalent growth rate reduction (**Figure 2**), specifically, 11.25 μM for *S. aureus* Newman and 15 μM for *S. epidermidis* Tü3298. The transcriptomes of each species were determined under challenge and control conditions using RNA-Seq, with resolution of the differentially expressed genes between these conditions.

### Sapienic Acid Challenge Transcriptomes

In response to sapienic acid challenge, *S. aureus* Newman showed 1224 significantly differentially expressed (DE) genes; 630 genes were upregulated and 594 were downregulated (**Supplementary Table S1A**). *S. epidermidis* Tü3298 showed 1505 significantly DE genes in response to sapienic acid challenge; 708 genes were upregulated and 797 were downregulated (**Supplementary Table S1B**). A greater proportion of DE genes across the genome was observed for *S. epidermidis* Tü3298 (64.5% of 2332 total genes) compared with *S. aureus* Newman (45.6% of 2686 total genes).

**FIGURE 3:**
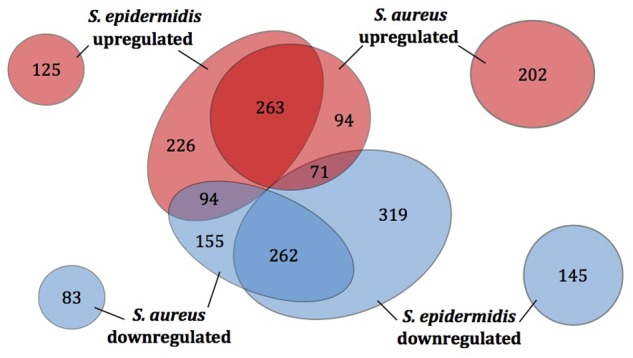
**Differentially expressed genes of *S. aureus* and *S. epidermidis* post challenge with sapienic acid.** The number of homologous genes of *S. aureus* Newman and *S. epidermidis* Tü3298 (central circles) that were upregulated (red) or downregulated (blue) following sapienic acid challenge were compared. Numbers of upregulated and downregulated genes with no homologue in the other species are shown by the outer circles.

Based on their homologous gene content, the sapienic acid transcriptomes of *S. aureus* and *S. epidermidis* were compared, which revealed 525 shared DE genes with a common regulation pattern (**Figure 3**, **Supplementary Table S1C**). Thus, the common transcriptomic response represents less than one half or one third of the DE genes of *S. aureus* and *S. epidermidis*, respectively. For both species, a selection of DE genes were confirmed using qPCR (**Supplementary Figure S1**).

**FIGURE 4:**
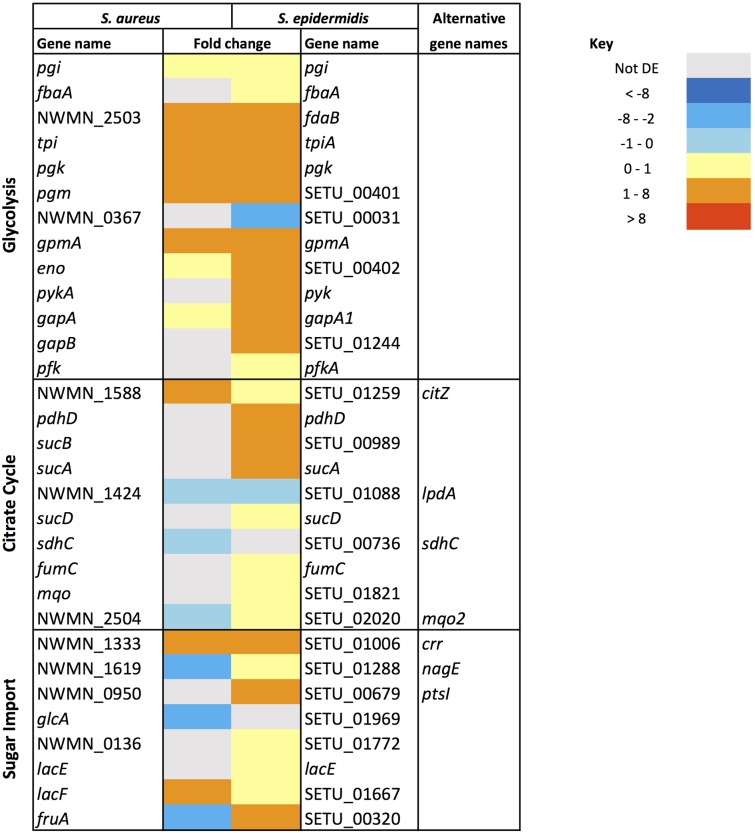
**Changes in gene expression of carbon metabolism associated genes in *S. aureus* and *S. epidermidis* challenged with sapienic acid**.

**FIGURE 5:**
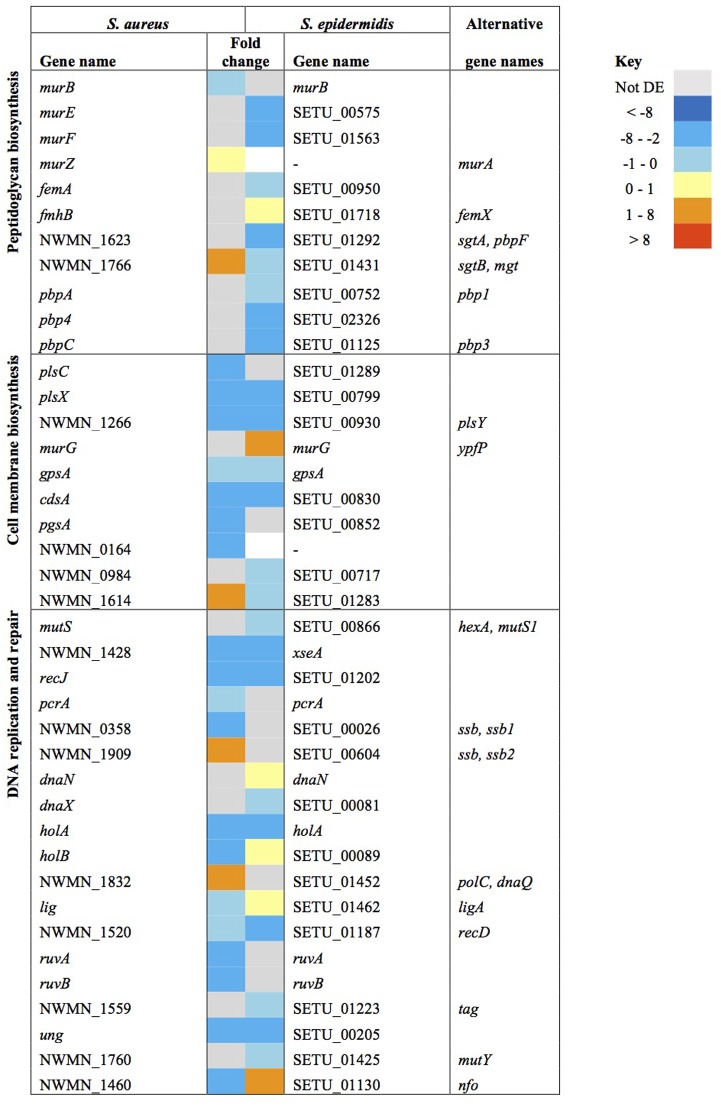
**Changes in gene expression of growth associated genes in *S. aureus* and *S. epidermidis* challenged with sapienic acid**.

Similarities in regulation within the shared responses to sapienic acid are likely to reflect a common response to the membrane depolarization mode of action that disrupts function of the electron transport chain (Cartron et al., 2014). Consistent with this mode of action, we identified upregulated transcription of genes required for sugar uptake, glycolysis, the TCA cycle, NADPH/NADP+ recycling and pyruvate metabolism (**Figure 4**). This shared response may enable the staphylococci to maintain ATP synthesis following disruption of energy generation at the membrane. Downregulated transcription was associated with genes for cell growth in both species (**Figure 5**), including peptidoglycan biosynthesis, cell membrane biosynthesis and DNA replication and repair. This response is consistent with adaptation of the bacteria to their changed environment, and with the growth lag observed following sapienic acid challenge of both *S. aureus* and *S. epidermidis* (**Figure 2**).

Downregulation of cell membrane glycerophospholipid biosynthesis genes, when combined with the upregulation of fatty acid degradation genes (*aldA*, NWMN_1858/SETU_01602, NWMN_2090/SETU_01661, NWMN_2091/SETU_01662), supports the description of sapienic acid incorporation into membrane lipids and lipoproteins in staphylococci (Parsons et al., 2012).

Host fatty acids are metabolized in *S. aureus* by a fatty acid kinase consisting of FakA plus FakB1 or FakB2 subunits in the phospholipid biosynthesis pathway (Parsons et al., 2014). The *fakA* and *fakB1* genes were downregulated in both *S. aureus* Newman and *S. epidermidis* Tü3298. Gene *fakB2* was upregulated only in *S. epidermidis*; since FakB2 binds long chain unsaturated fatty acids this supports sapienic acid incorporation into the cell phospholipid in this species. Incorporation of AFAs into cellular lipoproteins and phospholipids was suggested as a detoxification mechanism (Desbois and Smith, 2010), while incorporation of fatty acids into lipoproteins enhances the immune response against *S. aureus* (Nguyen et al., 2015).

### Sapienic Acid Transcriptome and Niche Colonization

The levels of topical skin lipids vary across the body surface (Lampe et al., 1983), and sebaceous richness inversely correlates with staphylococcal frequency (Costello et al., 2009; Coates et al., 2014). Cutaneous skin lipids, including sapienic acid, have potential to act as environmental cues for niche adaptation, particularly with the extensive sapienic acid-dependent transcription changes observed that indicate major transcriptome reprofiling in both staphylococci studied here.

Such an environmentally responsive pathway could correspond to the reduced virulence factor expression mediated by SaeRS in response to sapienic acid in *S. aureus*, which may promote its colonization over infection (Neumann et al., 2015). *S. aureus* Newman used in this study has an *saeS* mutation, resulting in constitutive expression of the SaeRS two-component system regulon (Cue et al., 2015). This mutation likely explains the lack of differential expression of hemolysins in the *S. aureus* Newman data set. The *saeRS* operon homologs in *S. epidermidis* (SETU_00325 and SETU_00326, respectively) were not differentially expressed in response to sapienic acid. Moreover, genes of the *S. epidermidis* SaeRS regulon (Handke et al., 2008) did not show any particular expression pattern that would suggest this regulon was modulated. On this basis, we propose that SaeRS is not a key regulator of the sapienic acid survival response of *S. epidermidis*.

Sapienic acid responsive gene expression changes included determinants that protect staphylococci from innate immune defenses of the skin. Adhesin genes implicated in colonization of the nose and skin, such as *sdrC* in *S. aureus* Newman and *ebh* genes in *S. epidermidis* Tü3298, were upregulated (7.4 and 1.3–2.6 fold, respectively) after challenge (**Supplementary Table S1**). In response to sapienic acid *S. aureus* Newman markedly upregulated (2.8–52.5 fold) capsule biosynthesis genes, approximating the response that we previously reported of *S. aureus* MRSA252 to linoleic acid (Kenny et al., 2009). Despite this pronounced upregulation of capsule biosynthesis genes, Neumann et al. (2015) identified that capsule deficient mutants do not have altered sapienic acid survival, at least for the laboratory strain SH1000 they studied.

Comparing each species’ response to sapienic acid there is further distinction between two-component signal transduction systems. The sapienic acid response of *S. epidermidis* Tü3298 includes upregulation of the *vraD* and *vraE* which encode an ABC transporter, important for bacitracin resistance. Contrastingly *vraDE* is not differentially expressed in *S. aureus* Newman. The VraDE transporter is regulated by the GraRS two-component system, that responds to cationic antimicrobial peptides (Pietiainen et al., 2009). Resistance to cationic antimicrobial peptides is an important factor in skin colonization and marks out a further distinction between the responses of each species to sapienic acid, with the potential for a coordinated antimicrobial response in *S. epidermidis*.

### Sapienic Acid Transcriptomes and Resistance

Comparison of the sapienic acid transcriptomes of *S. aureus* and *S. epidermidis* revealed candidate genes that might be associated with sapienic acid resistance based upon their upregulation or presence in a pathway. The contribution of these genes to resistance was explored with allelic replacement mutants.

**FIGURE 6:**
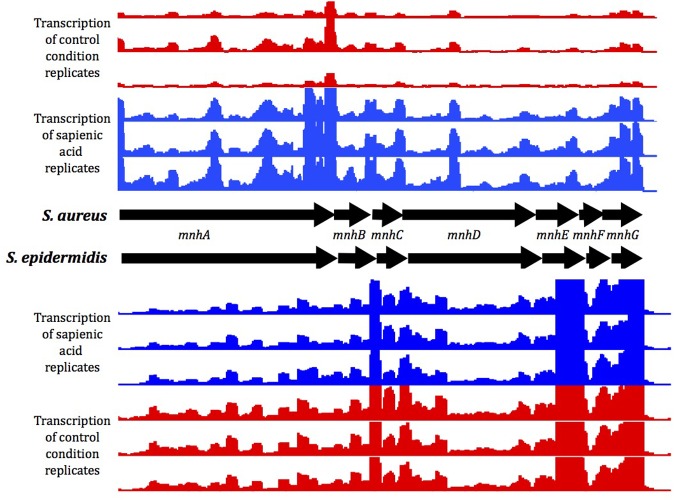
**Gene expression of the *mnh* operon under control conditions (red) or following sapienic acid challenge (blue).** Mapped reads for replicate RNA-Seq data were visualized in integrated genome browser (IGB).

Genes of the *mnhABCDEFG* operon were all considerably upregulated in *S. aureus* (4.7–6.8 fold change), though less upregulated in *S. epidermidis* (1.3–1.9 fold change) after sapienic acid challenge. Analysis of transcript abundance data (**Figure 6**) revealed that expression of the *mnh* operon was high in *S. epidermidis* during normal growth (control) conditions (232–1341.3 FPKM), while little expression was evident in *S. aureus* in these conditions (22.3–124.1 FPKM). A *mnhF* in-frame deletion mutant was investigated here for a role in sapienic resistance and had a twofold reduction in MIC (24 μg ml^-1^) compared with isogenic *S. aureus* SH1000 (48 μg ml^-1^). A recent study by Sannasiddappa et al. (2015) determined that *mnhF* confers resistance to bile salts through efflux of cholic acid. Several *mnh* genes encode Mrp family secondary antiporter proteins associated with cation/proton transport which can increase the transmembrane electrical potential in staphylococci (Swartz et al., 2007).

In addition to the *mnh* operon, multiple putative cation antiporters and osmoprotectant transporters were upregulated in response to sapienic challenge in *S. aureus* (NWMN_2457, NWMN_2050, NWMN_2089 and NWMN_0690) and *S. epidermidis* (SETU_02263 and SETU_00248-00254). The transcriptional upregulation of these transporters may protect the cell from the effects of solute leakage and membrane depolarization caused by sapienic acid (Greenway and Dyke, 1979; Parsons et al., 2012).

**FIGURE 7:**
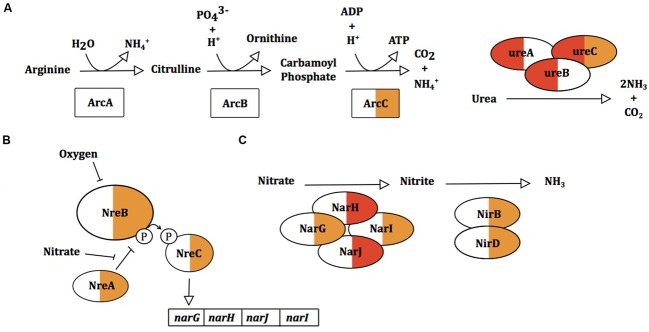
**Gene expression changes in ammonia and ammonium producing processes arginine deiminase pathway** **(A)**, urease activity **(B)** and the nitrate reductase pathway **(C)** in *S. aureus* and *S. epidermidis*. Proteins within pathways are colored for *S. aureus* Newman (left) and *S. epidermidis* Tü3298 (right) for changes in gene expression following sapienic acid challenge.

In response to sapienic acid challenge, *S. epidermidis* and *S. aureus* upregulate expression of different metabolic pathways that generate ammonia. *S. aureus* upregulated the urease operon (12.2–13.4 fold) with no consistent differential expression of this operon in *S. epidermidis* (**Figure 7**). In contrast, *S. epidermidis* upregulated *arcC* (1.7-fold) of the arginine deiminase pathway while the *arc* operon was not DE in *S. aureus. S. epidermidis* also upregulated the oxygen-responsive NreABC nitrogen regulation system (1.9–2.5 fold) and likewise upregulated the nitrate and nitrite reduction pathways (1.6–3.6 and 1–2.2 fold, respectively). Nitrate and nitrite dissimilation is coupled to the generation of a proton motive force in anoxic conditions and nitrite dissimilation generates cytoplasmic ammonia (Schlag et al., 2008). The *arc*, nitrate and nitrite reductase operons are induced in staphylococci only in the absence of oxygen (Fedtke et al., 2002; Lindgren et al., 2014; Nilkens et al., 2014), further supporting that there is reduced uptake or altered perception of oxygen following sapienic acid challenge. *S. epidermidis* using nitrate as an alternative acceptor for its electron transport chain might offer considerable metabolic flexibility compared with *S. aureus*.

**FIGURE 8:**
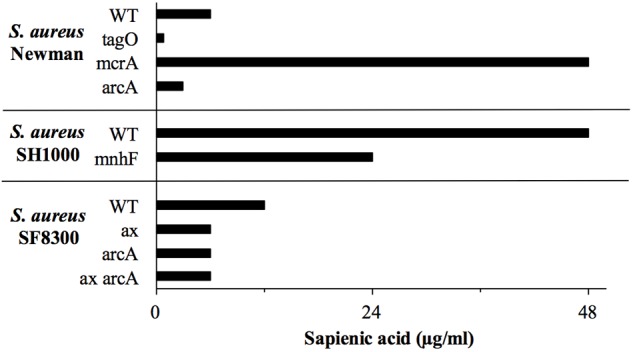
**Sapienic acid MICs of *S. aureus* wild type and mutant strains.** MICs were determined using the broth microdilution method. MICs are the lowest concentration found to inhibit growth. Mutants of genes predicted to be important in sapienic acid resistance due to RNA Seq data were contructed or obtained.

We previously determined that expression of the arginine deiminase pathway operon (*arcABC*) contributes to *S. aureus* linoleic acid resistance (Kenny et al., 2009). The arginine deiminase pathways encoded chromosomally or on the ACME element *arc* both result in ammonia production, so here we tested if either of these operons contribute to sapienic acid resistance. The sapienic acid MIC was determined for *S. aureus* SF8300 and its isogenic ACME element deletion mutant, SF8300ax; the MIC of the mutant was twofold lower than its parent strain (**Figure 8**). The sapienic acid MIC of the chromosomal *arcA* mutants of *S. aureus* SF8300 and Newman were also twofold lower than their parent strains. While there was no difference in the MIC of SF8300ax and SF8300ax *arcA* mutant, there was a consistent reduction in growth of the SF8300ax *arcA* mutant compared with SF8300ax (at 3 μg/ml sapienic acid in the MIC assay mean OD_600=_ 0.41 and 0.94, respectively) (**Figure 8**).

Staphylococcal myosin cross reactive antigen (McrA) homologs were previously proposed as antimicrobial lipid resistance determinants (Coates et al., 2014) due to their similarity with fatty acid hydratases of streptococci (Bevers et al., 2009; Volkov et al., 2010; Rosberg-Cody et al., 2011; Joo et al., 2012). Following sapienic acid challenge there was increased expression of the *mcrA* gene homologs (NWMN_0050 and SETU_00673), 7.4 and 5.4-fold, respectively in *S. aureus* and *S. epidermidis*. Fatty acid hydratase enzymes convert unsaturated fatty acids into their saturated counterparts, which is a detoxification mechanism for oleic acid in *Streptococcus pyogenes* (Volkov et al., 2010). The staphylococcal McrA has no obvious secretion motifs making it unlikely to act on extracellular sapienic acid, though it could act to facilitate its metabolism intracellularly. Here, allelic replacement of *mcrA* in *S. aureus* was achieved to investigate its contribution to resistance. Somewhat unexpectedly, the sapienic acid MIC of *S. aureus* Newman *mcrA* (*NWMN_0050*) was greater (48 μg ml^-1^) than its isogenic parent strain (6 μg ml^-1^) (**Figure 8**). This increased resistance phenotype was reversed in the Newman *mcrA* pSK5632+*mcrA* complementation strain, which had the same MIC as the wild type. Mutation of *mcrA* takes the survival of *S. aureus* Newman to a level of sapienic acid survival similar to a *S. epidermidis* strain. This indicates that an inability to saturate sapienic acid to palmitic acid through McrA acitivity, or a distinct cellular lipid conversion by McrA impacts *S. aureus* survival. By comparison, the *mcrA* gene (SETU_006730) of *S. epidermidis* Tü3298 contains a premature stop codon, indicating this gene activity may not be functional.

Wall teichoic acid (WTA) deficient mutants have reduced MIC for antimicrobial fatty acids (Kohler et al., 2009) and here, *S. aureus* Newman *tagO* was shown to have a very reduced sapienic acid MIC (< 0.8 μg ml^-1^), over eight times lower than its isogenic wild type strain (**Figure 8**). Despite its importance for survival, genes of the WTA biosynthesis pathway were downregulated in both species (*tagA*, *tagG*, *dltX*, and *gtaB* in *S. aureus*, *tagB* and *tagF*/*tarF* in *S. epidermidis*). In addition, both *S. epidermidis* and *S. aureus* downregulated *dlt*, *mprF*, and *isdA* genes which would be predicted to increase cell hydrophobicity, but these transcription changes may reflect peptidoglycan modification genes mirroring a reduction in cell wall biosynthesis during a period of reduced growth post sapienic acid challenge.

### *S. epidermidis* Specific Resistance Determinants

Key sapienic acid resistance determinants that differentiate the increased *S. epidermidis* sapienic acid MIC from *S. aureus* might be identifiable from their signature of transcription upregulation and/or presence only in the *S. epidermidis* data set. Of those 38 genes upregulated >2-fold in *S. epidermidis* with no homolog in *S. aureus*, 12 are annotated with transport functions (**Supplementary Table S2**). These genes may counteract the leakage of solutes caused by sapienic acid (Greenway and Dyke, 1979; Parsons et al., 2012); transport of osmolytes is a key resistance mechanism used by staphylococci during acid stress, where there is also accompanying membrane depolarization (Bore et al., 2007). A further nine genes of the 38 were hypothetical genes, revealing a need to evaluate potential resistance determinants within this gene set.

## Conclusion

*S. epidermidis* strains have greater sapienic resistance than *S. aureus*. The transcriptional responses of *S. aureus* and *S. epidermidis* to sapienic acid reveal that in addition to a shared stimulon, there are multiple distinct pathways modulated in each species. Our data identifies potential roles for the use of alternative respiration pathways, ammonia production and cation/osmolyte transport in differential survival from sapienic acid.

## Author Contributions

JM designed and performed experiments and wrote the paper. JA designed and performed experiments. MH conceived and designed experiments and wrote the paper. All authors read and approved submission.

## Conflict of Interest Statement

The authors declare that the research was conducted in the absence of any commercial or financial relationships that could be construed as a potential conflict of interest.

## Footnotes

1 http://www.ebi.ac.uk/arrayexpress

## References

[B1] Allgaier H. Jung G. Werner R. Schneider U. Zahner H. (1986). Epidermin - sequencing of a heterodet tetracyclic 21-peptide amide antibiotic. *Eur. J. Biochem.* 160 9–22. 10.1111/j.1432-1033.1986.tb09933.x 3769923

[B2] Arikawa J. Ishibashi M. Kawashima M. Takagi Y. Ichikawa Y. Imokawa G. (2002). Decreased levels of sphingosine, a natural antimicrobial agent, may be associated with vulnerability of the stratum corneum from patients with atopic dermatitis to colonization by *Staphylococcus aureus*. *J. Investig. Dermatol.* 119 433–439. 10.1046/j.1523-1747.2002.01846.x 12190867

[B3] Bera A. Herbert S. Jakob A. Vollmer W. Gotz F. (2005). Why are pathogenic staphylococci so lysozyme resistant? The peptidoglycan OM acetyltransferase OatA is the major determinant for lysozyme resistance of *Staphylococcus aureus*. *Mol. Microbiol.* 55 778–787. 10.1111/j.1365-2958.2004.04446.x 15661003

[B4] Bevers L. Pinkse M. Verhaert P. Hagen W. (2009). Oleate hydratase catalyzes the hydration of a nonactivated carbon-carbon bond. *J. Bacteriol.* 191 5010–5012. 10.1128/JB.00306-09 19465645 PMC2715725

[B5] Bieber T. (2008). Mechanisms of disease: Atopic dermatitis. *N. Engl. J. Med.* 358 1483–1494. 10.1056/NEJMra074081 18385500

[B6] Bore E. Langsrud S. Langsrud O. Rode T. Holck A. (2007). Acid-shock responses in *Staphylococcus aureus* investigated by global gene expression analysis. *Microbiology* 153 2289–2303. 10.1099/mic.0.2007/005942-0 17600073

[B7] Cartron M. England S. Chiriac A. Josten M. Turner R. Rauter Y. (2014). Analysis of the bactericidal activity of the human skin fatty acid, cis-6-hexadecanoic acid on *Staphylococcus aureus*. *Antimicrob. Agents Chemotherapy* 58 3599–3609. 10.1128/AAC.01043-13 PMC4068517 24709265

[B8] Cho J. Xuan C. Miller L. (2010). Lucky number seven: RNase 7 can prevent *Staphylococcus aureus* skin colonization. *J. Investig. Dermatol.* 130 2703–2706. 10.1038/jid.2010.294 21068735

[B9] Christensen G. Simpson W. Bisno A. Beachey E. (1982). Adherence of slime-producing strains of *Staphylococcus epidermidis* to smooth surfaces. *Infect. Immun.* 37 318–326. 6179880 10.1128/iai.37.1.318-326.1982 PMC347529

[B10] Christensen G. Simpson W. Younger J. Baddour L. Barrett F. Melton D. (1985). Adherence of coagulase negative staphylococci to plastic tissue culture plates: a quantitative model for the adherence of staphylococci to medical devices. *J. Clin. Microbiol.* 22 966–1006. 10.1128/jcm.22.6.996-1006.1985 PMC271866 3905855

[B11] Coates R. Moran J. Horsburgh M. (2014). Staphylococci: colonizers and pathogens of human skin. *Future Microbiol.* 9 75–91. 10.2217/fmb.13.145 24328382

[B12] Costello E. Lauber C. Hamady M. Fierer N. Gordon J. Knight R. (2009). Bacterial community variation in human body habitats across space and time. *Science* 326 1694–1697. 10.1126/science.1177486 19892944 PMC3602444

[B13] Cue D. Junecko J. Lei M. Blevins J. Smeltzer M. Lee C. (2015). SaeRS-dependent inhibition of biofilm formation in *Staphylococcus aureus* Newman. *PLoS ONE* 10:e0123027 10.1371/journal.pone.0123027 PMC4390220 25853849

[B14] Davis K. Stewart J. Crouch H. Florez C. Hospenthal D. (2004). Methicillin-resistant *Staphylococcus aureus* (MRSA) nares colonization at hospital admission and its effect on subsequent MRSA infection. *Clin. Infect. Dis.* 39 776–782. 10.1086/422997 15472807

[B15] Desbois A. Smith V. (2010). Antibacterial free fatty acids: activities, mechanisms of action and biotechnological potential. *Appl. Microbiol. Biotechnol.* 85 1629–1642. 10.1007/s00253-009-2355-3 19956944

[B16] Diep B. Stone G. Basuino L. Graber C. Miller A. Des Etages S. (2008). The arginine catabolic mobile element and staphylococcal chromosomal cassette mec linkage: convergence of virulence and resistance in the USA300 clone of methicillin-resistant *Staphylococcus aureus*. *J. Infect. Dis.* 197 1523–U35. 10.1086/587907 18700257

[B17] Duquenne M. Fleurot I. Aigle M. Darrigo C. Borezée-Durant E. Derzelle S. (2010). Tool for quantification of staphylococcal enterotoxin gene expression in cheese. *Appl. Environ. Microbiol.* 76 1367–1374. 10.1128/AEM.01736-09 20061456 PMC2832361

[B18] Duthie E. Lorenz L. (1952). Staphylococcal coagulase: mode of action and antigenicity. *J. Gen. Microbiol.* 6 95–107. 10.1099/00221287-6-1-2-95 14927856

[B19] Fedtke I. Kamps A. Krismer B. Gotz F. (2002). The nitrate reductase and nitrite reductase operons and the narT gene of *Staphylococcus carnosus* are positively controlled by the novel two-component system NreBC. *J. Bacteriol.* 184 6624–6634. 10.1128/JB.184.23.6624-6634.2002 12426351 PMC135434

[B20] Fischer C. Drake D. Dawson D. Blanchette D. Brogden K. Wertz P. (2012). Antibacterial activity of sphingoid bases and fatty acids against Gram positive and Gram negative bacteria. *Antimicrob. Agents Chemother.* 56 1157–1161. 10.1128/AAC.05151-11 22155833 PMC3294957

[B21] Greenway D. Dyke K. (1979). Mechanism of the inhibitory action of linoleic acid on the growth of *Staphylococcus aureus*. *J. Gen. Microbiol.* 115 233–245. 10.1099/00221287-115-1-233 93615

[B22] Handke L. D. Rogers K. L. Olson M. E. Somerville G. A. Jerrells T. J. Rupp M. E. (2008). Staphylococcus epidermidis *saeR* is an effector of anaerobic growth and a mediator of acute inflammation. *Infect. Immun.* 76 141–152. 10.1128/IAI.00556-07 17954724 PMC2223648

[B23] Higaki S. Morohashi M. Yamagishi T. Hasegawa Y. (1999). Comparative study of staphylococci from the skin of atopic dermatitis patients and from healthy subjects. *Int. J. Dermatol.* 38 265–269. 10.1046/j.1365-4362.1999.00686.x 10321941

[B24] Holden M. Feil E. Lindsay J. Peacock S. Day N. Enright M. (2004). Complete genomes of two clinical *Staphylococcus aureus* strains: evidence for the rapid evolution of virulence and drug resistance. *PNAS* 101 9786–9791. 10.1073/pnas.0402521101 15213324 PMC470752

[B25] Horsburgh M. Aish J. White I. Shaw L. Lithgow J. Foster S. (2002). Sigma B modulates virulence determinant expression and stress resistance: characterization of a Functional rsbU strain derived from *Staphylococcus aureus* 8325-4. *J. Bacteriol.* 184 5457–5467. 10.1128/JB.184.19.5457-5467.2002 12218034 PMC135357

[B26] Horsburgh M. Wiltshire M. Crossley H. Ingham E. Foster S. (2004). PheP, a putative amino acid permease of *Staphylococcus aureus*, contributes to survival in vivo and during starvation. *Infect. Immun.* 72 3073–3076. 10.1128/IAI.72.5.3073-3076.2004 15102825 PMC387907

[B27] Horsburgh M. J. Clements M. O. Crossley H. Ingham E. Foster S. J. (2001). PerR controls oxidative stress resistance and iron storage proteins and is required for virulence in *Staphylococcus aureus*. *Infect. Immun.* 69 3744–3754. 10.1128/IAI.69.6.3744-3754.2001 11349039 PMC98383

[B28] Joo Y. Jeong K. Yeom S. Kim Y. Kim Y. Oh D. (2012). Biochemical characterization and FAD-binding analysis of oleate hydratase from *Macrococcus caseolyticus*. *Biochimie* 94 907–915. 10.1016/j.biochi.2011.12.011 22203098

[B29] Kanehisa M. Goto S. (2000). KEGG: Kyoto encyclopedia of genes and genomes. *Nucleic Acids Res.* 28 27–30. 10.1093/nar/28.1.27 10592173 PMC102409

[B30] Kanehisa M. Goto S. Sato Y. Furumichi M. Tanabe M. (2012). KEGG for integration and interpretation of large-scale molecular data sets. *Nucleic Acids Res.* 40 D109–D114. 10.1093/nar/gkr988 22080510 PMC3245020

[B31] Kelly J. (2013). *Genomic and Metagenomic Analysis of the Skin Microbiota.* Ph.D. thesis, University of Liverpool, Liverpool.

[B32] Kenny J. Moran J. Kolar S. Ulanov A. Li Z. Shaw L. (2013). Mannitol utilisation is required for protection of *Staphylococcus aureus* from human skin antimicrobial fatty acids. *PLoS ONE* 8:e67698 10.1371/journal.pone.0067698 PMC3701532 23861785

[B33] Kenny J. Ward D. Josefsson E. Jonsson I. Hinds J. Rees H. (2009). The *Staphylococcus aureus* response to unsaturated long-chain free fatty acids: survival mechanisms and virulence implications. *PLoS ONE* 4:e4344 10.1371/journal.pone.0004344 PMC2629846 19183815

[B34] Kluytmans J. Wertheim H. (2005). Nasal carriage of *Staphylococcus aureus* and prevention of nosocomial infections. *Infection* 33 3–8. 10.1007/s15010-005-4012-9 15750752

[B35] Kluytmans J. Vanbelkum A. Verbrugh H. (1997). Nasal carriage of *Staphylococcus aureus*: epidemiology, underlying mechanisms, and associated risks. *Clin. Microbiol. Rev.* 10 505–520. 9227864 10.1128/cmr.10.3.505 PMC172932

[B36] Kohler T. Weidenmaier C. Peschel A. (2009). Wall teichoic acid protects *Staphylococcus aureus* against antimicrobial fatty acids from human skin. *J. Bacteriol.* 191 4482–4484. 10.1128/JB.00221-09 19429623 PMC2698495

[B37] Kong H. Oh J. Deming C. Conlan S. Grice E. Beatson M. (2012). Temporal shifts in the skin microbiome associated with disease flares and treatment in children with atopic dermatitis. *Genome Res.* 22 850–859. 10.1101/gr.131029.111 22310478 PMC3337431

[B38] Kreiswirth B. Lofdahl S. Betley M. Oreilly M. Schlievert P. Bergdoll M. (1983). The toxic shock syndrome exotoxin structural gene is not detectably transmitted by a prophage. *Nature* 305 709–712. 10.1038/305709a0 6226876

[B39] Lampe M. Burlingame A. Whitney J. Williams M. Brown B. Roitman E. (1983). Human stratum corneum lipids- characterization and regional variations. *J. Lipid Res.* 24 120–130. 6833889

[B40] Langmead B. Trapnell C. Pop M. Salzberg S. (2009). Ultrafast and memory-efficient alignment of short DNA sequences to the human genome. *Genome Biol.* 10:R25 10.1186/gb-2009-10-3-r25 PMC2690996 19261174

[B41] Li M. Diep B. Villaruz A. Braughton K. Jiang X. Deleo F. (2009). Evolution of Virulence in epidemic community-associated methicillin-resistant *Staphylococcus aureus*. *PNAS* 106 5883–5888. 10.1073/pnas.0900743106 19293374 PMC2667066

[B42] Libberton B. (2011). *The Ecology of Staphylococcus aureus.* Ph.D. thesis, University of Liverpool, Liverpool.

[B43] Lindgren J. Thomas V. Olson M. Chaudhari S. Nuxoll A. Schaeffer C. (2014). Arginine deiminase in *Staphylococcus epidermidis* functions to augment biofilm maturation through pH homeostasis. *J. Bacteriol.* 196 2277–2289. 10.1128/JB.00051-14 24727224 PMC4054176

[B44] Mack D. Siemssen N. Laufs R. (1992). Parallel induction by glucose of adherence and a polysacharide antigen specific for plastic-adherent-Staphylococcus-epidermidis - evidence for functional relation to intercellular-adhesion. *Infect. Immun.* 60 2048–2057. 1314224 10.1128/iai.60.5.2048-2057.1992 PMC257114

[B45] Moran G. Krishnadasan A. Gorwitz R. Fosheim G. Linda M. Mcdougal L. (2006). Methicillin-resistant *S. aureus* infections among patients in the emergency department. *N. Engl. J. Med.* 355 666–674. 10.1056/NEJMoa055356 16914702

[B46] Moran J. Horsburgh M. (2016). Whole-genome sequence of *Staphylococcus epidermidis* Tü3298. *Genome Announc.* 4 e112–e116. 10.1128/genomeA.00112-16 PMC4786668 26966218

[B47] Moss B. Squire J. (1948). Nose and skin carriage of *Staphylococcus aureus* in patients receiving penicillin. *Lancet* 1 320–325. 10.1016/S0140-6736(48)92088-1 18905395

[B48] Nagarajan V. Elasri M. (2007). SAMMD: *Staphylococcus aureus* microarray meta-database. *BMC Genomics* 8:351 10.1186/1471-2164-8-351 PMC2117023 17910768

[B49] Neumann Y. Ohlsen K. Donat S. Engelmann S. Kusch H. Albrecht D. (2015). The effect of skin fatty acids on *Staphylococcus aureus*. *Arch. Microbiol.* 197 245–267. 10.1007/s00203-014-1048-1 25325933 PMC4326651

[B50] Nguyen M. Hanzelmann D. Härtner T. Peschel A. Götz F. (2015). Skin-specific unsaturated fatty acids boost the *Staphylococcus aureus* innate immune response. *Infect. Immun.* 84 205–215. 10.1128/IAI.00822-15 26502910 PMC4693987

[B51] Nilkens S. Koch-Singenstreu M. Niemann V. Gotz F. Stehle T. Unden G. (2014). Nitrate/oxygen co-sensing by an NreA/NreB sensor complex of *Staphylococcus carnosus*. *Mol. Microbiol.* 91 381–393. 10.1111/mmi.12464 24261791

[B52] Nolan T. Hands R. Bustin S. (2006). Quantification of mRNA using real-time RT-PCR. *Nature Protocols* 1 1559–1582. 10.1038/nprot.2006.236 17406449

[B53] Parsons J. Broussard T. Bose J. Rosch J. Jackson P. Subramanian C. (2014). Identification of a two-component fatty acid kinase responsible for host fatty acid incorporation by *Staphylococcus aureus*. *Proc. Natl. Acad. Sci. U.S.A.* 111 10532–10537. 10.1073/pnas.1408797111 25002480 PMC4115530

[B54] Parsons J. Yao J. Frank M. Jackson P. Rock C. (2012). Membrane disruption by antimicrobial fatty acids releases low-molecular-weight proteins from *Staphylococcus aureus*. *J. Bacteriol.* 194 5294–5304. 10.1128/JB.00743-12 22843840 PMC3457211

[B55] Pietiainen M. Francois P. Hyyrylainen H. Tangomo M. Sass V. Sahl H. (2009). Transcriptome analysis of the responses of *Staphylococcus aureus* to antimicrobial peptides and characterization of the roles of vraDE and vraSR in antimicrobial resistance. *BMC Genomics* 10:429 10.1186/1471-2164-10-429 PMC2748101 19751498

[B56] Robinson M. Mccarthy D. Smyth G. (2010). EdgeR: a Bioconductor package for differential expression analysis of digital gene expression data. *Bioinformatics* 26 139–140. 10.1093/bioinformatics/btp616 19910308 PMC2796818

[B57] Robinson M. Oshlack A. (2010). A scaling normalization method for differential expression analysis of RNA-seq data. *Genome Biol.* 11 R25 10.1186/gb-2010-11-3-r25 PMC2864565 20196867

[B58] Rosberg-Cody E. Liavonchanka A. Gobel C. Ross R. O’sullivan O. Fitzgerald G. (2011). Myosin-cross-reactive antigen (MCRA) protein from Bifidobacterium breve is a FAD-dependent fatty acid hydratase which has a function in stress protection. *BMC Biochem* 12:9 10.1186/1471-2091-12-9 PMC3063827 21329502

[B59] Sannasiddappa T. Hood G. Hanson K. Costabile A. Gibson G. Clarke S. (2015). *Staphylococcus aureus* MnhF mediates cholate efflux and facilitates survival under human colonic conditions. *Infect. Immun.* 83 2350–2357. 10.1128/IAI.00238-15 25824834 PMC4432758

[B60] Schafer L. Kragballe K. (1991). Abnormalities in epidermal lipid-metabolism in patients with Atopic dermatitis. *J. Investig. Dermatol.* 96 10–15. 10.1111/1523-1747.ep12514648 1987285

[B61] Schlag S. Fuchs S. Nerz C. Gaupp R. Engelmann S. Liebeke M. (2008). Characterization of the oxygen-responsive NreABC regulon of *Staphylococcus aureus*. *J. Bacteriol.* 190 7847–7858. 10.1128/JB.00905-08 18820014 PMC2583599

[B62] Soares J. Lopes C. Tavaria F. Delgado L. Pintado M. (2013). A diversity profile from the staphylococcal community on atopic dermatitis skin: a molecular approach. *J. Appl. Microbiol.* 115 1411–1419. 10.1111/jam.12296 23910049

[B63] Swartz T. Ito M. Ohira T. Natsui S. Hicks D. Krulwich T. (2007). Catalytic properties of *Staphylococcus aureus* and Bacillus members of the secondary cation/proton antiporter-3 (Mrp) family are revealed by an optimized assay in an *Escherichia coli* host. *J. Bacteriol.* 189 3081–3090. 10.1128/JB.00021-07 17293423 PMC1855852

[B64] Takigawa H. Nakagawa H. Kuzukawa M. Mori H. Imokawa G. (2005). Deficient production of hexadecenoic acid in the skin is associated in part with the vulnerability of atopic dermatitis patients to colonization by *Staphylococcus aureus*. *Dermatology* 211 240–248. 10.1159/000087018 16205069

[B65] Volkov A. Liavonchanka A. Kamneva O. Fiedler T. Goebel C. Kreikemeyer B. (2010). Myosin cross-reactive antigen of Streptococcus pyogenes M49 encodes a fatty acid double bond hydratase that plays a role in oleic acid detoxification and bacterial virulence. *J. Biol. Chem.* 285 10353–10361. 10.1074/jbc.M109.081851 20145247 PMC2856241

[B66] Ye J. Coulouris G. Zaretskaya I. Cutcutache I. Rozen S. Madden T. (2012). Primer-BLAST: a tool to design target-specific primers for a polymerase chain reaction. *BMC Bioinfo*rmatics 13:134 10.1186/1471-2105-13-134 PMC3412702 22708584

