# Supplementary material for: Comparative Transcriptomics Reveals Discrete Survival Responses of S. aureus and S. epidermidis to Sapienic Acid
Source: Front Microbiol. 2017 Jan 25;8:33. doi: 10.3389/fmicb.2017.00033 (PMC5263133; doi:10.3389/fmicb.2017.00033)
Supplement: Supplementary file 3 [file Image_1.pdf]

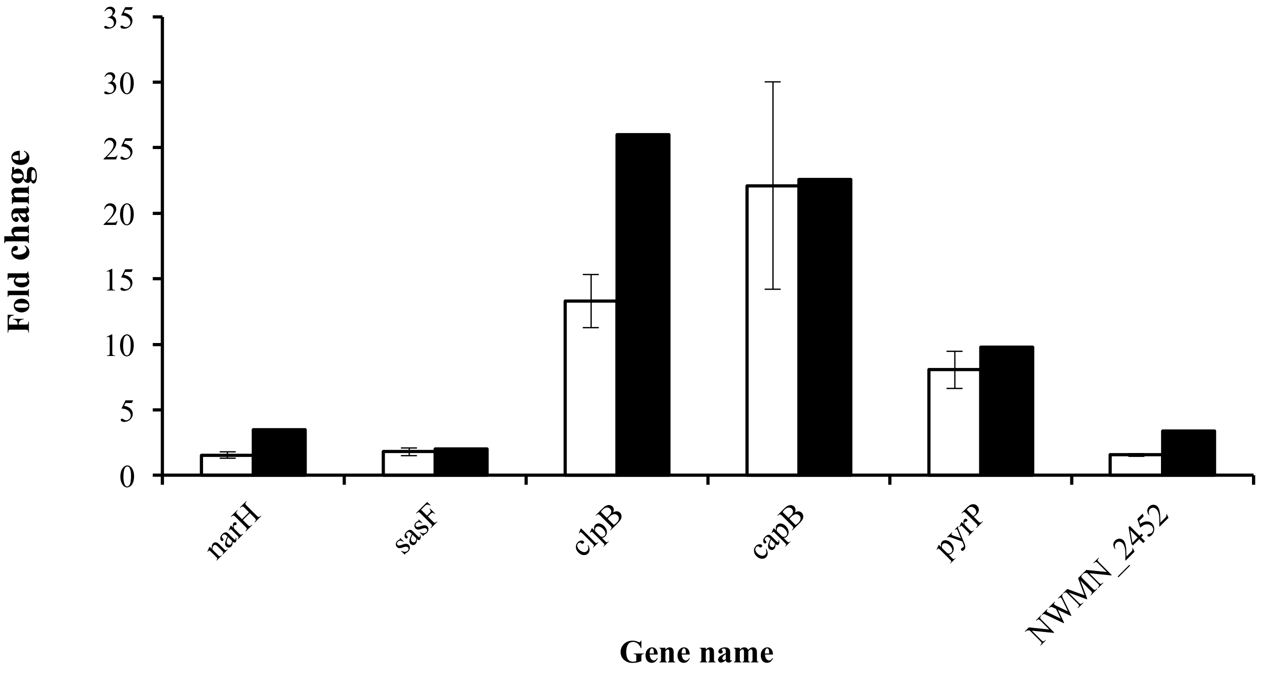

Supplementary Figure 1: Differential expression of *narH*, *sasF* and *clpB* in *S. epidermidis* and *capB*, *pyrP* and *farR* in *S. aureus* after challenge with sapienic acid, assessed by qPCR compared to RNA-Seq. Black bars indicate the fold change in gene expression from RNA-Seq data while white bars indicate fold change in gene expression from qPCR data.
